# Regulatory network of two circRNAs and an miRNA with their targeted genes under astilbin treatment in pulmonary fibrosis

**DOI:** 10.1111/jcmm.14550

**Published:** 2019-08-26

**Authors:** Guangping Lu, Jinjin Zhang, Xiangyong Liu, Wenbo Liu, Guohong Cao, Changjun Lv, Xiaoli Zhang, Pan Xu, Minge Li, Xiaodong Song

**Affiliations:** ^1^ Department of Clinical Nursing Binzhou Medical University Hospital Binzhou China; ^2^ Department of Cellular and Genetic Medicine, School of Pharmaceutical Sciences Binzhou Medical University Yantai China; ^3^ Department of Respiratory Medicine Binzhou Medical University Hospital Binzhou China

**Keywords:** astilbin, circRNAs, pulmonary fibrosis, RNA sequencing

## Abstract

Circular RNAs (circRNAs) are becoming new therapeutic drug targets. However, their profiles under astilbin treatment have not been reported yet. In this study, we analysed the global reprogramming of circRNA transcriptome and a regulatory network of circRNAs with their targeted genes under astilbin treatment in pulmonary fibrosis. A total of 145 circRNAs were differentially expressed in the astilbin‐treated group compared with the bleomycin‐treated group using RNA sequencing. In the bleomycin‐ and astilbin‐treated groups, 29 coexpressed circRNAs were found. The maximum number of circRNAs was distributed on chromosome two, and their length varieties were mainly within 1000 bp. Four differentially expressed circRNAs (circRNA‐662, 949, 394 and 986) were tested to validate the RNA sequencing data, and their targeted microRNAs and genes were analysed by qRT‐PCR, Western blot, Pearson correlation coefficient, a dual‐luciferase reporter system and anti‐AGO_2_ RNA immunoprecipitation. The results showed that circRNA‐662 and 949 can act as “miR‐29b sponges” targeting Gli2 and STAT3 to exert their functions. Our work suggests that the transcriptome complexity at the circRNA level under astilbin treatment. These circRNAs may be potential molecular targets for drug action.

## 1 INTRODUCTION

Pulmonary fibrosis is a progressive, dysregulated repair response to frequent lung injury. In the normal state, injury and repair are tightly regulated. However, in susceptible hosts, the injury will evoke an exaggerated repair response, leading to excess extracellular matrix production that causes extensive fibrogenesis.^1^ The fibrosis impairs the normal lung architecture, ultimately leading to its dysfunction and failure. Although considerable information is known, the mechanism that regulates fibrogenesis remains poorly understood. This situation hampers pulmonary fibrosis treatment.^2^, ^3^ Currently, drug treatments are still the main option for most pulmonary fibrosis patients, but the results have mostly been disappointing. Pirfenidone and nintedanib are examples of drugs used for treatment. However, despite their long‐term therapeutic efficacy, they produce side effects such as nausea, indigestion and hypertension.^4^, ^5^ Therefore, the identification of new and effective anti‐fibrotic agents with less adverse effects is an important research field in pulmonary fibrosis.

Astilbin has attracted considerable attention because of its anti‐inflammatory and anti‐oxidative properties.^6^, ^7^ It can alleviate psoriasis‐like skin lesions by inhibiting Th17‐related inflammation^8^ and ameliorate cisplatin‐induced nephrotoxicity by reducing ROS accumulation.^9^ In addition, astilbin can promote angiotensin‐converting enzyme inhibition in vitro^10^ and protect diabetic rat heart against ischaemia‐reperfusion injury.^11^ In a previous study, we reported that astilbin ameliorates fibrosis via blockade of the Hedgehog signalling pathway, in which we mainly focused on the role of coding RNAs under astilbin treatment.^12^ However, high‐throughput sequencing technology on RNA transcriptomes revealed that most of these transcripts are not translated to protein which are named as non‐coding RNAs (ncRNAs),^13^ such as microRNA (miRNA), long ncRNA (lncRNA) and circular RNA (circRNA). Moreover, increasing evidence revealed that these ncRNAs play diverse roles in the development and progression of human diseases and are becoming new therapeutic drug targets.^14^, ^15^, ^16^ However, the changes of ncRNAs under astilbin treatment have not been reported yet.

In our previous study, we found that ncRNAs play key roles in fibrosis and can be used as therapeutic targets for pulmonary fibrosis using microarray technology.^17^, ^18^, ^19^ However, microarray technology lacks sensitivity for low‐abundance RNA and may miss RNAs related to disease risk and progression. Moreover, compared with miRNA and lncRNA, circRNA is a novel class of RNA transcript and a new clinical diagnostic and prognostic biomarker.^20^, ^21^ Therefore, in this study, we first combined RNA sequencing and other experiments to analyse the global reprogramming of circRNA transcriptome under astilbin treatment in pulmonary fibrosis. We found that different types of RNAs can collaborate to establish a sophisticated regulatory network to exert their function. We hope by characterizing the interactions among mRNAs, miRNAs and circRNAs can enhance our understanding of disease pathogenesis and drug action, and create new theories for the pathogenesis and treatment of pulmonary fibrosis.

## 2 MATERIALS AND METHODS

### 2.1 Animal model

Mice with mean weight of 25 ± 2 g were purchased from the Model Animal Research Center of Nanjing University (Nanjing, China). Experiments on the animals studied were approved by the Committee on the Ethics of Animal Experiments of Binzhou Medical University. All animals were bred and maintained in a 12 hours light/dark cycle and allowed free access to food and water, and randomly divided into four groups: sham group, 40 mg/kg astilbin alone group (AST group), bleomycin‐treated group (BLM group) and BLM + 40 mg/kg AST‐treated group. BLM animal model was administered with 5 mg/kg BLM dissolved in saline via single intratracheal instillation under anaesthesia as previously described.^12^ At day 14, astilbin was orally administered every day. At day 30, all mice were killed, and lung tissue sections were collected and immediately frozen in liquid nitrogen for further studies.

### 2.2 Cell model and treatment

The mouse lung fibroblast cell line L929 was purchased from the Cell Bank of the Chinese Academy of Sciences. The cells were maintained in advanced minimum essential medium and supplemented with 10% newborn calf serum, 100 U/mL penicillin and 100 µg/mL streptomycin at 37°C under a humidified atmosphere of 5% CO_2_ and 95% air. The cells were first administered with 5 ng/mL transforming growth factor beta 1 (TGF‐β1) for 6 hours and then co‐treated with 40 µg/mL AST for 48 hours as previously described.^12^

### 2.3 Haematoxylin and eosin (H&E) and masson's trichrome staining

Lung tissues were fixed by instilling 4% paraformaldehyde overnight, dehydrated in 70% ethanol and embedded in paraffin wax. Transverse sections of 4 μm thickness were stained with H&E or Masson's trichrome following the manufacturer's standard protocol. Three sections collected from each lung were analysed in the experiment.

### 2.4 RNA sequencing

Total RNA was obtained from sham group, BLM‐treated group and astilbin‐treated group, respectively. mRNA was purified from total RNA using poly‐T oligo‐attached magnetic beads. Fragmentation was carried out using divalent cations under elevated temperature in NEBNext First Strand Synthesis Reaction Buffer (5×). These samples were used for sequencing. Sequencing libraries were generated using NEBNext^®^ Ultra^™^ RNA Library Prep Kit for Illumina^®^ (#E7530L, NEB) following the manufacturer's recommendations and index codes were added to attribute sequences to each sample. Following quantile normalization of the raw data, circRNA with at least two out of two present or marginal flags were selected for further data analysis. Differentially expressed circRNAs were identified through fold change filtering.

### 2.5 CircRNA identification pipeline

CircRNAs were predicted by CIRCexplorer.^22^ CIRCexplorer utilized the unmapped reads exported by TopHat and aligned these reads using TopHat‐Fusion.^23^ The alignment results were imported into CIRCexplorer to predict circRNAs. All circRNA candidates with junction reads <2 were discarded. The remaining circRNA candidates were considered bonafide circRNAs.

### 2.6 Gene ontology (GO) and Kyoto encyclopedia of genes and genomes (KEGG) analysis

The main functions of the target circRNAs were determined according to GO analysis. Pathway analysis was performed to determine the significant pathway of the differential genes according to KEGG. Fisher's exact test and chi‐squared were used to classify the GO category and select the significant pathway. The significance threshold was defined by *P*‐value and false discovery rate.

### 2.7 Quantitative real‐time reverse transcriptase polymerase chain reaction (qRT‐PCR)

Approximately 2 mg RNA was extracted from lung tissue samples for the synthesis of first‐strand cDNA. qRT‐PCR analysis was performed with the Rotor‐Gene 3000 Real‐Time PCR System with the following reaction conditions: predenaturation at 95°C for 30 seconds and PCR amplification for 35 cycles at 95°C for 15 seconds and at 60°C for 25 seconds. GAPDH was used as endogenous controls in each sample. By convention, changes in expression were determined using the 2^−ΔΔCT^ method for samples in the Rotor‐Gene 6 Software. Primers used in qRT‐PCR are shown in Table 1.

**Table 1:** Primers used in qRT‐PCR

Name	Sequence
circRNA‐394	
sense	ACAGCAGCAGCATCAACAGC
anti‐sense	CTTCTCTTACTGGTTCCCTGTCC
circRNA‐662	
sense	TCGAAGCAGAGACAGGACGC
anti‐sense	CTCAATGAAAGTCCTTATATCCGAG
circRNA‐949	
sense	GCATTAGTATAGTGGAATGGAAACC
anti‐sense	CTCTTGCATAGTTTGCCTCAAGT
circRNA‐986	
sense	AGCCCAACAAGTGTACGGTCC
anti‐sense	GGATGAAAGTGAAGGGAAAGC

### 2.8 Western blot

20 mg protein was subjected to 10% sodium dodecyl sulphate polyacrylamide gel electrophoresis, transferred onto polyvinylidene difluoride membranes and blocked with 5% non‐fat milk in Tris‐buffered saline and Tween‐20 (TBST; 50 mmol/L Tris‐HCl [pH 7.6], 150 mmol/L NaCl, 0.1% Tween‐20). Membranes were washed thrice with TBST buffer and incubated at 4°C overnight with specific antibodies. After washing with TBST, membranes were incubated with horseradish peroxidase‐labelled IgG for 1.5 hours. Membranes were then washed with TBST, incubated with ECL reagent and exposed. Then, membranes were subsequently stripped and re‐probed with glyceraldehyde 3‐phosphate dehydrogenase antibody (GAPDH), which served as loading control.

### 2.9 Statistical analysis

Data are expressed as the mean ± standard deviation (SD), and statistical significance was assessed by one‐way ANOVA with Student‐Newman‐Keuls post‐test for experiments comparing three or more groups. Probability values of <0.05 were considered significant. Statistical analyses were performed using SPSS version 19.0 software.

## 3 RESULTS

### 3.1 AST ameliorated pulmonary fibrosis in vivo and in vitro

**Figure 1:**
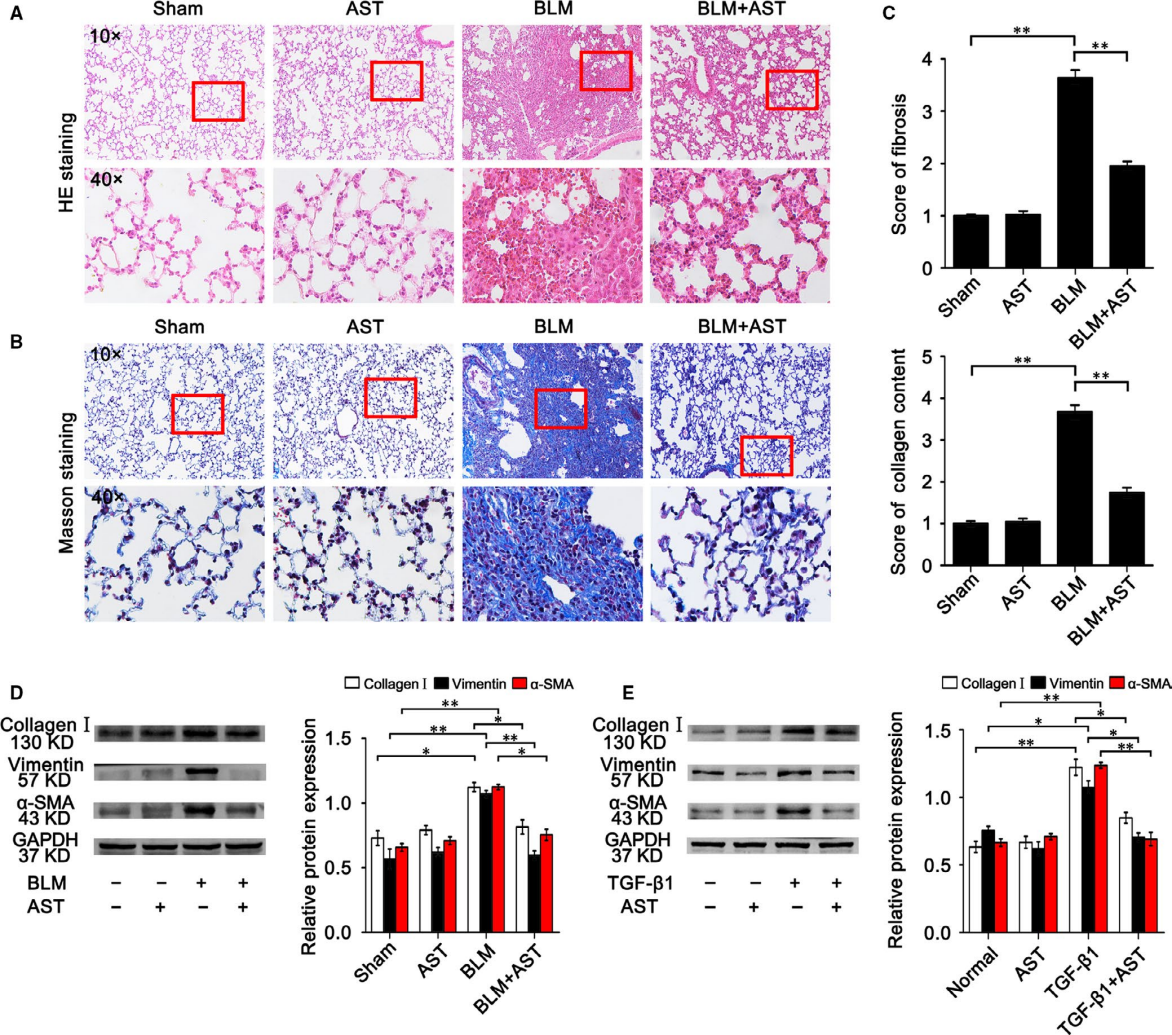
Identification of animal model. A, Lung tissue structure was observed with H&E staining. B, Collagen content (blue) was observed with Masson's trichrome staining. These photographs are representatives. C, Grade of lung fibrosis and collagen content. D, Western blot results showed that AST inhibited the expression of collagen, vimentin and a‐SMA in mice model. E, Western blot results showed that AST inhibited the expression of collagen, vimentin and a‐SMA in cell model. Each bar represents the mean ± SD, n = 6, **P < .01

To assess circRNA expression under astilbin treatment, H&E and Masson's trichrome staining were first used to evaluate the fibrosis model. H&E staining indicated that the alveoli from the sham group had clear hollow cavities with alveolar walls thinner than those in other groups. The BLM group had the thickest alveolar walls, and the hallmark of the fibroblastic foci was distinctly present among all groups. Compared with the BLM group, tissue sections had thinner alveolar walls and the histological characteristics of fibrosis had shown some improvement in the AST‐treated group (Figure 1A). Masson's trichrome staining showed that the collagen matrix and fibrosis lesions with cordlike distribution increased in the BLM group (Figure 1B). Scores of lung fibrosis and collagen were quantified according to H&E and Masson staining (Figure 1C). In addition, the results of Western blot experiments further confirmed that AST significantly ameliorated the fibrosis hallmarks of collagen, vimentin and a‐SMA in mice and cell models (Figure 1D,E).

### 3.2 Profiles of differentially expressed circRNAs

**Figure 2:**
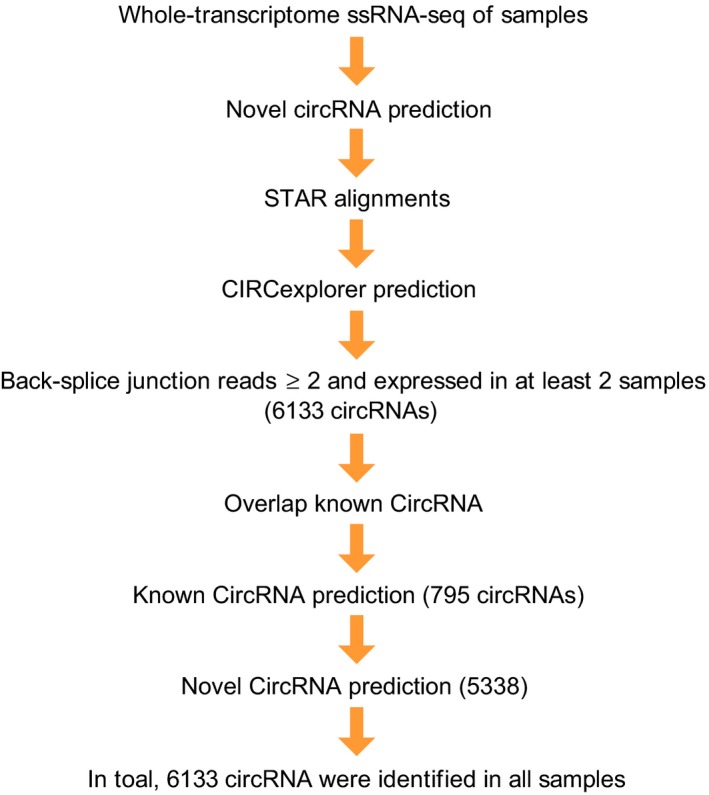
CircRNA identification pipeline

**Figure 3:**
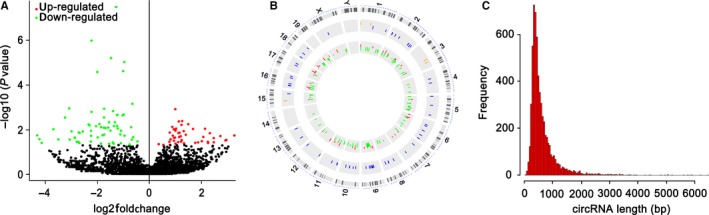
Differentially expressed circRNAs. A, Heat map of circRNAs. Fifty‐eight circRNAs were up‐regulated (red), and 87 circRNAs were down‐regulated (green) in the AST‐treated group compared with the BLM‐treated group. B, Distribution of differentially expressed circRNAs on chromosome. Most of them were mainly distributed on chromosome 2. C, Lengths of differentially expressed circRNAs. Most of them were within 1000 bp

A computational pipeline for the systematic identification of circRNAs is shown in Figure 2. The results of heat map analysis showed that the circRNAs are differentially expressed. A total of 145 circRNAs were differentially expressed in the AST‐treated group compared with the BLM‐treated group. Of these circRNAs, 58 were up‐regulated, and 87 were down‐regulated (Figure 3A). Then, we further analysed the distribution of these circRNAs on chromosomes. The circRNAs were distributed on chromosomes 1‐19. The maximum number of circRNAs was mainly distributed on chromosome two (Figure 3B). Their length varieties widely varied, and most of them were within 1000 bp (Figure 3C).

### 3.3 Analysis of the differentially expressed circRNAs' host genes

**Figure 4:**
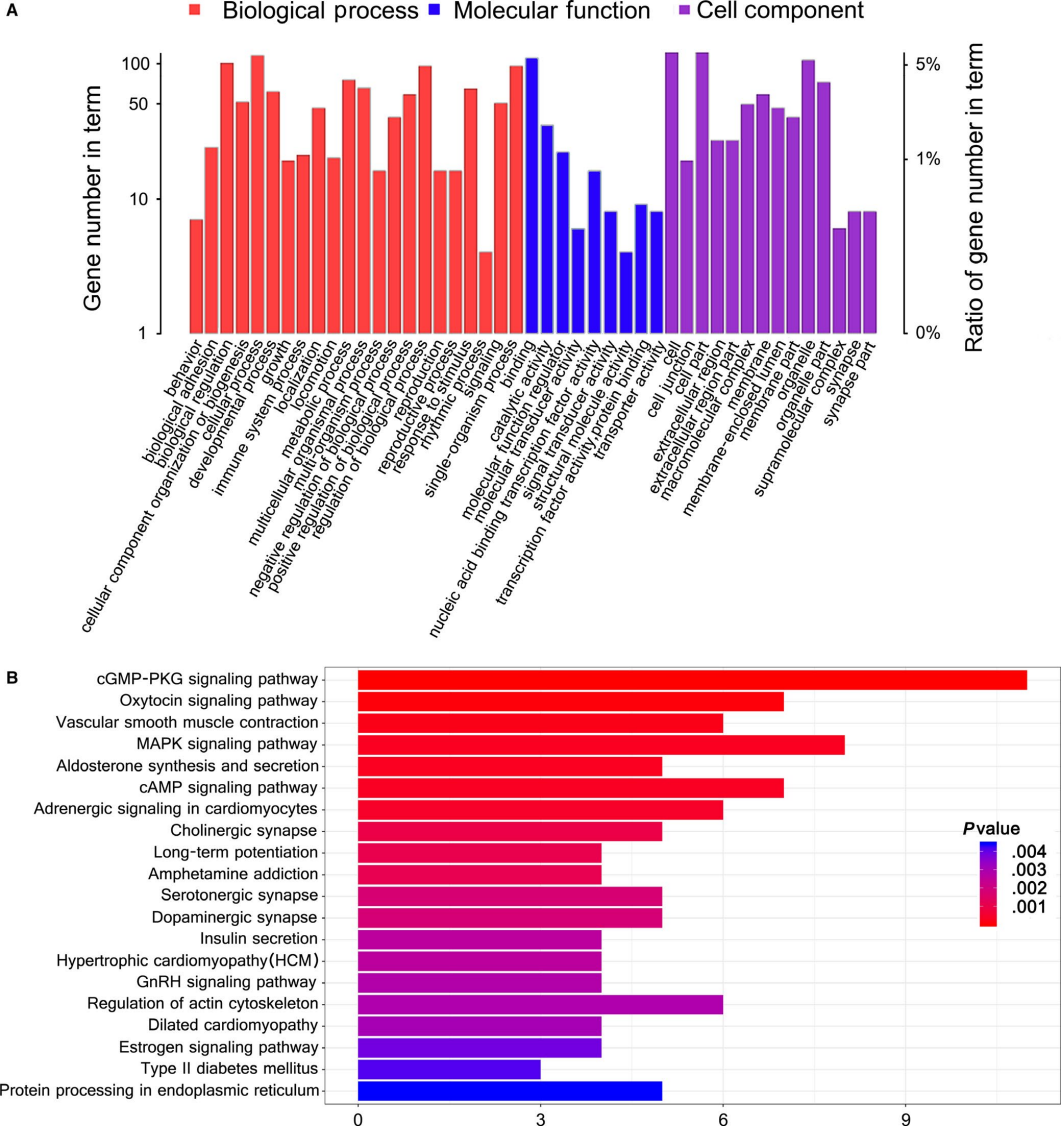
Analysis of circRNAs' host gene function. A, Biological process, molecular function, and cell component of these host genes were analysed using the GO database. B, Biological pathways of these host genes were analysed using the KEGG database

One of circRNA mechanisms is that circRNA exert its regulatory function by specifically interacting with its host genes. Therefore, we analysed the host genes of differentially expressed circRNAs by using GO and KEGG analysis. According to the GO annotation tool, these host genes were mainly enriched for GO terms related to biological adhesion, developmental process, cell junction, extracellular region, signal transducer activity and structural molecule activity (Figure 4A). Biological pathways associated with the host genes of differentially expressed circRNAs were analysed using the KEGG database. These pathways were associated with cGMP‐PKG, MAPK and vascular smooth muscle contraction (Figure 4B).

### 3.4 Analysis of differentially expressed circRNAs' targeted genes

The other circRNA mechanism is that circRNA act as “miRNA sponges” to regulate the targeted genes, namely ceRNA. Regulatory network of two circRNAs and an miRNA with their targeted genes under astilbin treatment was explored in detail.

**Figure 5:**
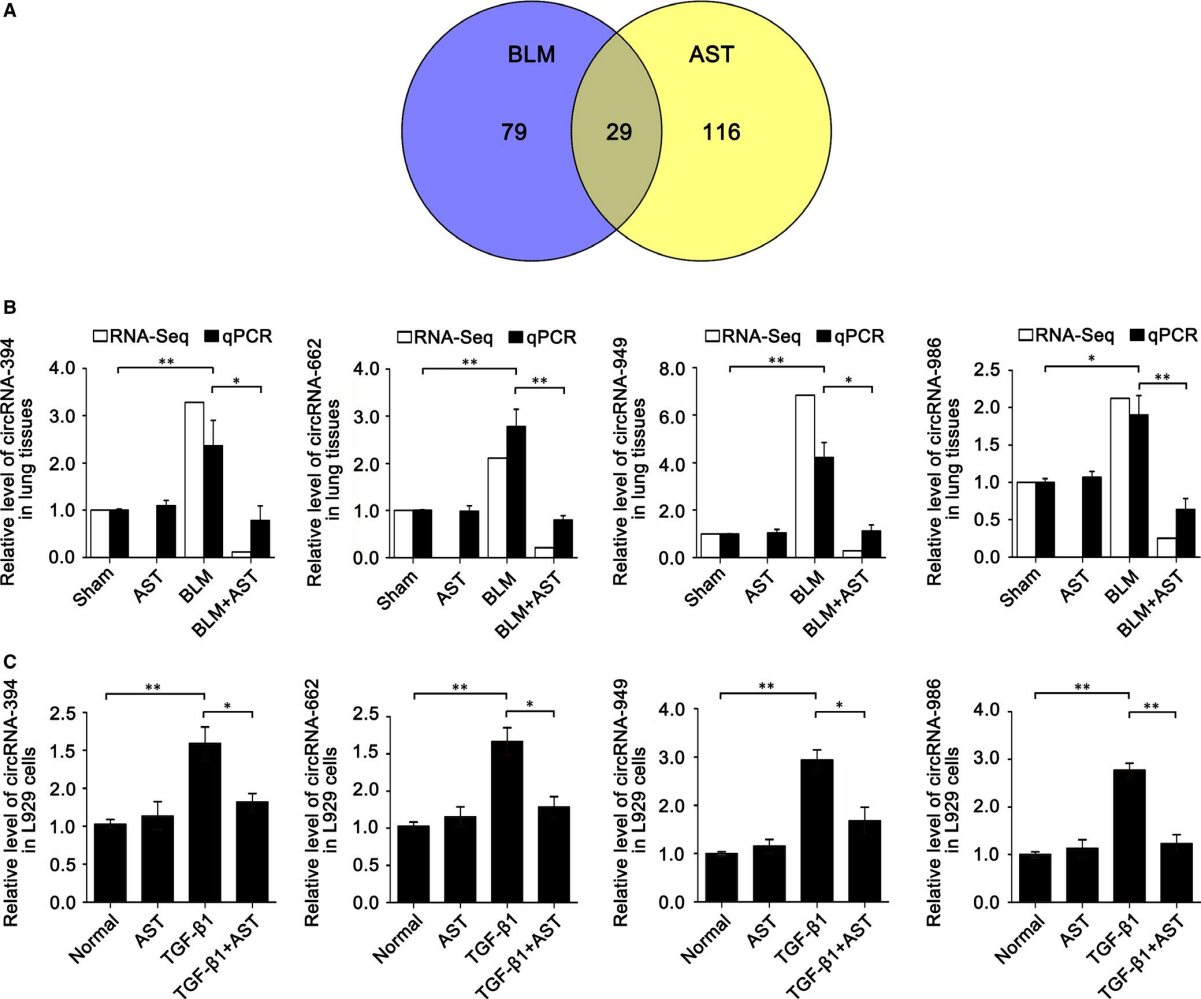
Analysis of differentially coexpressed circRNAs. A, Venn diagram indicates 29 differentially coexpressed circRNAs among BLM and AST groups. B, Expression of circRNA‐394, circRNA‐662, circRNA‐949 and circRNA‐986 by using RNA sequencing and qRT‐PCR in mice model. C, Expression of circRNA‐394, circRNA‐662, circRNA‐949 and circRNA‐986 by qRT‐PCR in cell model. Each bar represents the mean ± SD, n = 6, *P < .05, **P < .01

**Figure 6:**
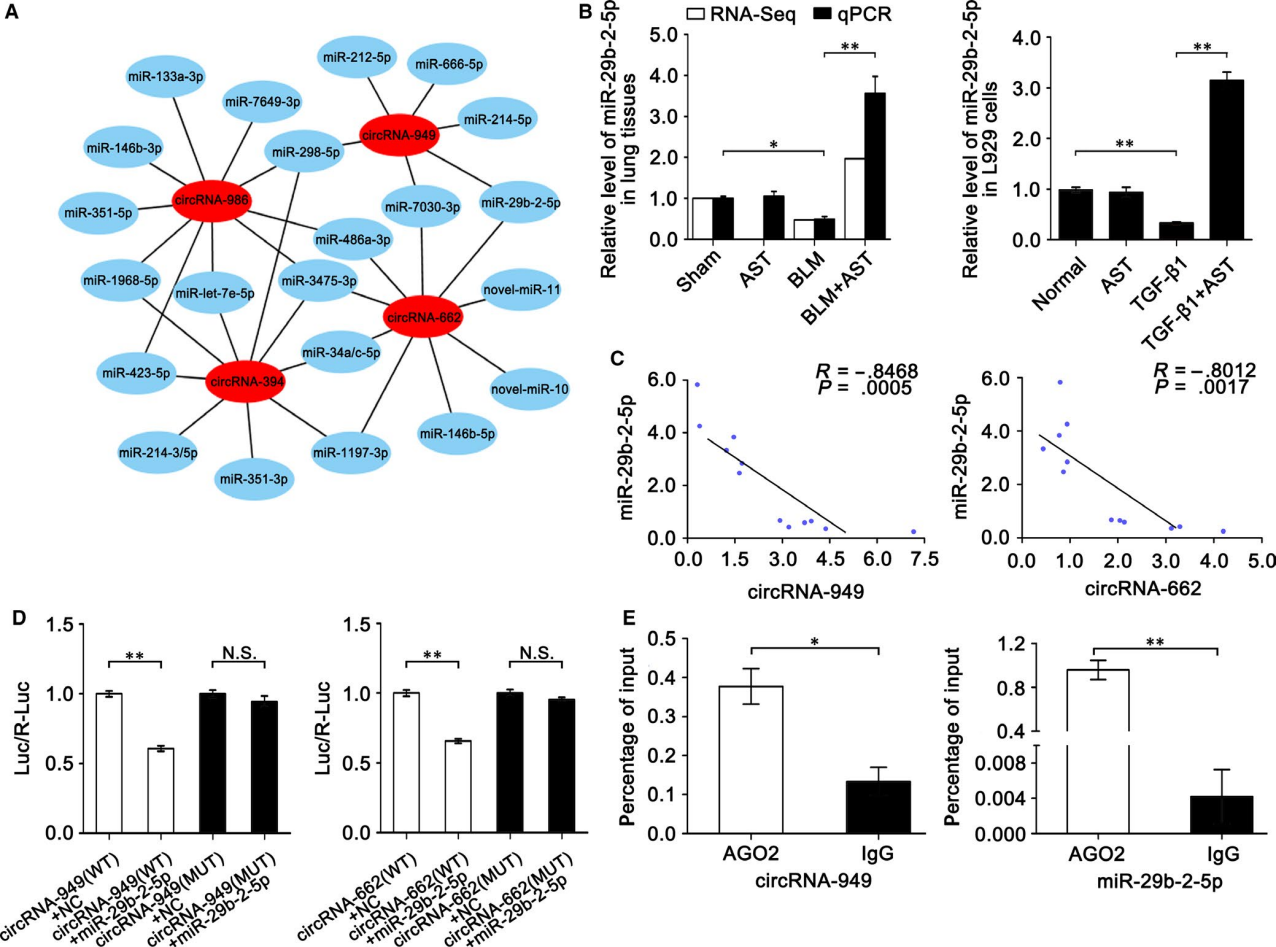
Expression of the targeted miR‐29b‐2‐5p. A, Binding sites of circRNA‐394, circRNA‐662, circRNA‐949 and circRNA‐986 for miRNAs. B, Expression of miR‐29b‐2‐5p by using RNA sequencing and qRT‐PCR in vivo and in vitro. C, Inverse relationship of circRNA‐949 and circRNA‐662 with miR‐29b‐2‐5p was analysed by Pearson correlation coefficient. D, CircRNA‐949 and circRNA‐662 are the direct targets of miR‐29b‐2‐5p by using a dual‐luciferase reporter system experiment. E, RIP data confirmed the result of the dual‐luciferase experiment. Each bar represents the mean ± SD, n = 6, *P < .05, **P < .01

Firstly, we analysed the differentially coexpressed circRNAs between BLM vs sham group and AST vs BLM group to validate the RNA‐sequencing data. In total, 29 differentially coexpressed circRNAs were obtained in the BLM and AST groups (Figure 5A). Then, we randomly selected four circRNAs (circRNA‐394, 662, 949 and 986) from 29 circRNAs and determined their expression levels through qRT‐PCR. All data are in agreement with RNA‐sequencing data (Figure 5B). CircRNAs can act as “miRNA sponges” to exert their functions; therefore, we further analysed the four circRNA binding sites for miRNAs. As previously described,^24^ miRNA binding site prediction in circRNAs was based on their full‐length sequence. Twenty‐two miRNAs were found to bind to circRNA‐394, 662, 949 and 986. Among these miRNAs, circRNAs‐949 and 662 sponged miR‐29b‐2‐5p (Figure 6A) and had higher binding scores than others. Therefore, miR‐29b‐2‐5p was selected for further investigation. RNA‐sequencing and qRT‐PCR results showed that miR‐29b‐2‐5p increased under astilbin treatment compared with BLM treatment (Figure 6B). Its expression was inversely correlated with circRNAs‐949 and 662 (Figure 6C). A dual‐luciferase reporter system (Figure 6D) and anti‐AGO_2_ RNA immunoprecipitation (RIP) were conducted (Figure 6E) to confirm the identity of their direct target. The results showed that both circRNAs‐949 and 662 are the direct targets of miR‐29b‐2‐5p.

**Figure 7:**
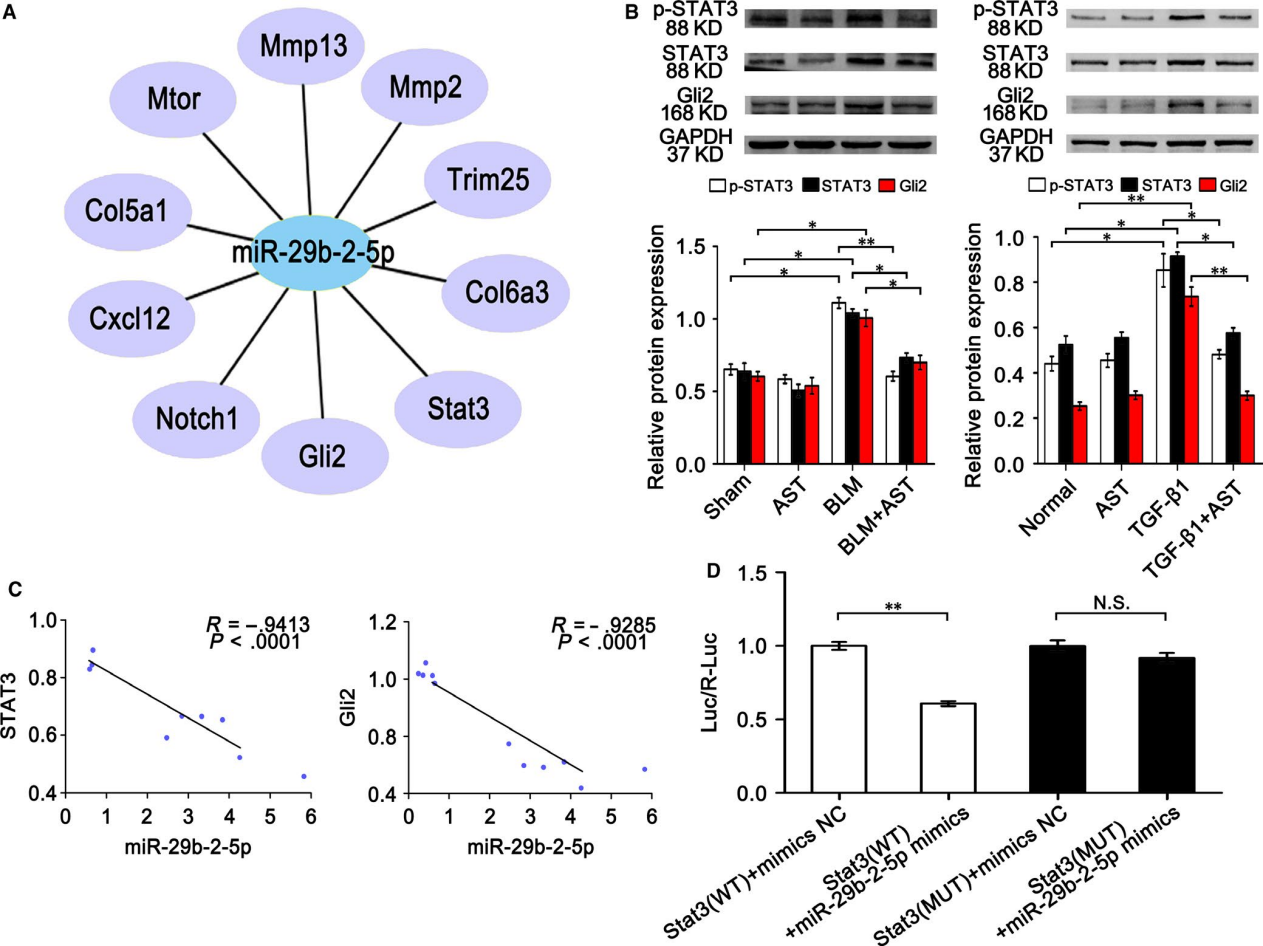
Expression of the targeted genes STAT3 and Gli2. A, MiR‐29b‐2‐5p targeted genes were predicted. B, Western blot analysis showed that STAT3 and Gli2 protein levels decreased after astilbin treatment. C, Inverse relationships of miR‐29b‐2‐5p with STAT3 and Gli2 were analysed by Pearson correlation coefficient. D, The result of the dual‐luciferase experiment showed that STAT3 was the direct target gene of miR‐29b‐2‐5p. Each bar represents the mean ± SD, n = 6, *P < .05, **P < .01

Therefore, the miR‐29b‐2‐5p targeted genes were predicted based on TargetScan, MiRanda data and miRBase (Figure 7A). Among the miR‐29b‐2‐5p targeted genes, we focused on STAT3 and Gli2 because they are the main regulatory factors of lung fibrosis in our previous study.^12^, ^18^ STAT3 and Gli2 decreased under astilbin treatment compared with BLM treatment (Figure 7B). Their expressions were inversely correlated with miR‐29b‐2‐5p (Figure 7C). A dual‐luciferase reporter system was conducted to confirm the identity of their direct target. The results showed that the results showed that STAT3 is the direct target of miR‐29b‐2‐5p (Figure 7D).

## 4 DISCUSSION

In this study, we described 145 differentially expressed circRNAs and analysed the relationships between these circRNAs and their protein‐coding genes under astilbin treatment in pulmonary fibrosis. We found that (a) circRNAs have important functions in regulating gene expression in pulmonary fibrosis and affect drug activity and that (b) various types of RNAs and their protein‐coding genes can collaborate to establish a sophisticated regulatory network.

With the development of high‐throughput sequencing, emerging evidence has indicated that ncRNAs play key roles in fibrosis. To date, most studies on the role of ncRNAs during pulmonary fibrogenesis have focused on miRNAs and lncRNAs, which regulate mRNA translation via the RNA interference pathway.^25^ Pandit et al reported that let‐7d inhibition is a key regulatory event in preventing lung fibrosis.^26^ Jiang et al summarized the role of lncRNAs in the process of lung fibrosis.^27^ However, the occurrence of circRNAs in fibrosis remains largely unknown. CircRNAs are derived from transcripts that are back‐spliced and joined head to tail at the splice sites.^28^, ^29^ Their covalently closed ring structure make them highly stable, even exceeding the stability of the housekeeping gene GAPDH.^30^ Bachmayr‐Heyda et al discovered a negative correlation of circRNA abundance and proliferation in lung fibrosis.^31^ Chao et al found that circHECTD1 promotes silica‐induced pulmonary fibrosis via HECTD1.^32^, ^33^ A recent study from our laboratory based on a circRNA microarray analysis identified 67 significantly dysregulated circRNAs in IPF patients, indicating the fundamental roles of circRNAs in pathological processes of lung fibrosis.^19^ In this study, 145 differentially expressed circRNAs were identified under astilbin treatment using RNA sequencing.

CircRNAs have complex regulatory mechanisms.^34^ For example, they can segregate RNA binding proteins^35^, ^36^and even be translated into proteins.^37^, ^38^ In addition, circRNAs can act as miRNA sponges to affect the targeted mRNA, called competing endogenous RNAs.^20^ Although not all circRNAs can act as competing endogenous RNAs,^39^, ^40^ the possibility of circRNAs acting as miRNA sponges cannot be ignored.^20^ For example, CircHIPK3 contains two binding sites for miR‐558 and sponges miR‐558 to suppress heparanase expression in bladder cancer.^41^ CircHRCR can act as an endogenous miR‐223 sponge to inhibit cardiac hypertrophy and heart failure.^42^ However, circRNAs acting as miRNA sponges in lung fibrosis have rarely been reported. By contrast, lncRNAs such as lncCHRF, n341773 and H19 contribute to lung fibrosis by acting as miRNA sponges.^27^ Based on our previous studies, competing endogenous RNAs are present not only in lncRNAs but also in circRNAs.^19^, ^43^ In this study, we showed the existence of competing endogenous RNAs under drug action. CircRNA‐662 and 949 can function as miR‐29b sponges to regulate STAT3 and Gli2, which are the main regulatory factors of lung fibrosis. STAT3 is up‐regulated in patients with lung fibrosis. Inhibition of STAT3 expression protects mice from BLM‐induced lung fibrosis.^18^, ^44^ Gli2 is one of the components of the Hedgehog signalling pathway. In our previous work, we analysed the function of this pathway in pulmonary fibrosis under astilbin action.^12^ Kleaveland et al recently reported that three different classes of ncRNAs converge in a regulatory network whereby an lncRNA represses an miRNA via target‐directed miRNA degradation, which in turn enables the accumulation of a circRNA in the mouse brain.^45^ This finding has once again proved that various types of ncRNAs and their protein‐coding genes can collaborate to regulate gene expression. It also indicates that silencing multiple abnormally expressed genes simultaneously is important to enhance the efficacy of disease treatments. Based on this consideration, Li designed an artificial lncRNA, which simultaneously interferes with multiple miRNAs.^46^

RNA sequencing is an approach to perform transcriptome profiling. Many studies reported the usefulness of RNA sequencing as a deep‐sequencing technology for the detection of potential biomarkers or candidate therapeutic targets in many diseases. Craciun et al identified CDH11, MRC1 and PLTP as biomarkers of kidney fibrosis by RNA sequencing.^47^ Lee et al analysed the transcriptome complexity through RNA sequencing in normal and failing murine hearts.^48^ However, few studies have identified the profiles of differentially expressed circRNAs under drug action using RNA sequencing in progressive lung fibrosis. Our findings regarding the crosstalk and molecular consequences of these circRNAs provide candidate therapeutic targets for drug treatment. From a clinical perspective, the targeting of circRNAs as a novel therapeutic approach will require a deeper understanding of their function and mechanism of action. However, in the short term, changes in circRNA expression are likely to be used as biomarkers for disease stratification and/or assessment of drug action.

## CONFLICT OF INTEREST

The authors declare no conflicts of interest.

## AUTHOR CONTRIBUTIONS

XD Song, ME Li, P Xu, JJ Zhang and CJ Lv participated in the conception, hypothesis and design of the study. GP Lu and WB Liu performed the experiments. GH Cao, XY Liu and XL Zhang carried out the statistical analyses. All authors contributed to interpretation of the data. XD Song wrote the manuscript and all authors made critical revisions. All authors read and approved the final manuscript.

## Data Availability

The analysed data sets generated during the study are available from the corresponding author on reasonable request.
